# Pancytopenia as a Consequence of Sepsis and Intravenous Antibiotic Drug Toxicity

**DOI:** 10.7759/cureus.3994

**Published:** 2019-02-01

**Authors:** Jake N Cho, Stephen Avera, Kenneth Iyamu

**Affiliations:** 1 Internal Medicine, Ocala Regional Medical Center/ University of Central Florida College of Medicine, Ocala, USA

**Keywords:** pancytopenia, agranulocytosis, vancomycin, drug toxicity, anemia, sepsis, disseminated intravascular coagulation

## Abstract

This case involves a 62-year-old male with a prior history of epidural abscess and L1-L2 osteodiscitis who was admitted because of low back pain. The patient was previously treated for methicillin-susceptible Staphylococcus aureus (MSSA) discitis in the L1/L2 vertebral region with intravenous (IV) nafcillin through a peripherally inserted central catheter (PICC). However, he returned after four months with recurrent low back pain along with chills and fever. He was admitted for severe sepsis related to the L1-L2 region osteomyelitis and discitis.

The Infectious Disease department initially started the patient on IV vancomycin and cefepime; however, routine labs on the second day of IV antibiotics showed concern for pancytopenia with white blood cell count (WBC) decreased to 2.5 thou/mm^3^, Hgb to 6.2 g/dL, Hct to 20.8%, and platelets to 82 thou/mm^3^ from baseline values of WBC 3.9 thou/mm^3^, Hgb 8.3 g/dL, Hct 28%, and platelets 126 thou/mm^3^. Due to concern for pancytopenia in the setting of severe sepsis, extensive hematologic workup was pursued to evaluate for disseminated intravascular coagulation (DIC) and bone marrow suppression. The patient also had a positive fecal occult blood test, so the Gastroenterology department was consulted for esophagogastroduodenoscopy (EGD) and colonoscopy. Furthermore, despite appropriate outpatient treatment for MSSA osteodiscitis, the patient was bacteremic with Staphylococcus aureus. Hence, the Cardiology department was consulted to rule out cardiac valvular vegetation.

This case presents a unique case of pancytopenia involving elements of drug-induced aplastic anemia as well as DIC-related sepsis. The agranulocytosis may have been a consequence of drug reaction to IV vancomycin. The anemia and thrombocytopenia may have been caused by DIC. Repeat computed tomography (CT) guided spinal aspiration confirmed pan-sensitive Staphylococcus aureus infection of the L1/L2 vertebral region. Treatment was reverted to nafcillin monotherapy and fortunately his hematologic function normalized, avoiding the need for advanced treatments such as intravenous immunoglobulin infusion therapy (IVIG) or high dose steroids.

## Introduction

Acquired agranulocytosis is a rare condition with a reported incidence ranging from one to five cases per million population per year. An association with medications can be found in two-thirds or more of the incidents [1]. Neutropenia is usually a result of decreased production or increased destruction. There are a number of medications implicated as potential causes of neutropenia or agranulocytosis, the most definitive medications being those that cause bone marrow suppression. In this case report, we describe the observation of vancomycin-associated agranulocytosis as well as hemolytic/aplastic anemia. Published case reports have cited vancomycin-induced neutropenia or thrombocytopenia separately, but very few have reported pancytopenia in the setting of sepsis with or without drug toxicity.

## Case presentation

**Figure 1:**
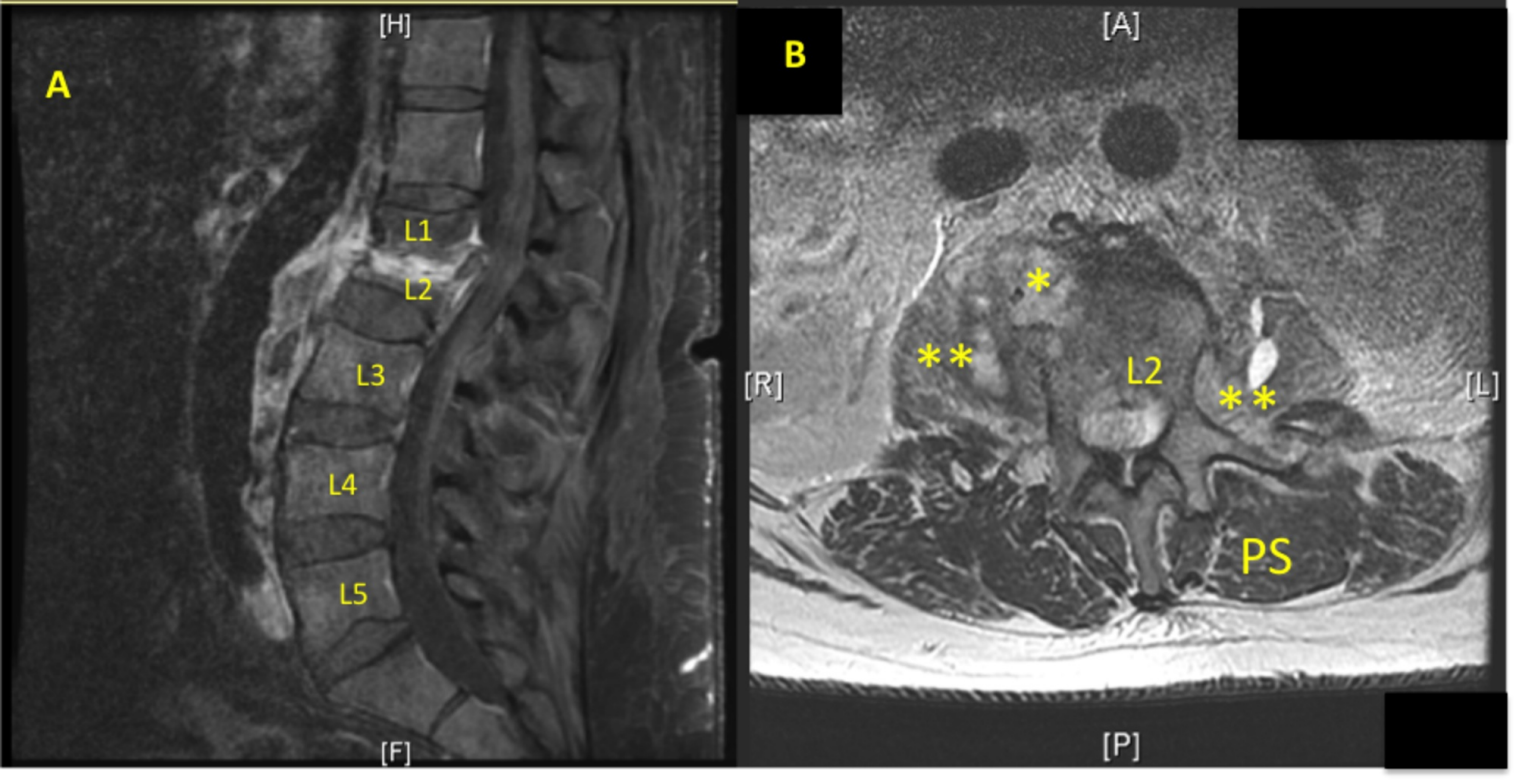
Initial Admission MRI Lumbar Initial admission, Figure 1A (MRI T1 w contrast, sagittal): Compression fracture at L2 with retropulsion of the posterior-superior margin. Bone destruction particularly involving the inferior aspect of the L1 vertebra and portions of the L2 vertebra representing discitis and osteomyelitis. Figure 1B (MRI T2 wo contrast, axial): Paravertebral phlegmon (yellow *) extending into left and right psoas muscles (double yellow *) with fluid signal intensity suggesting small abscesses. Moderate ventral thecal sac compression and moderate spinal stenosis. Paraspinal muscles (PS).

**Figure 2:**
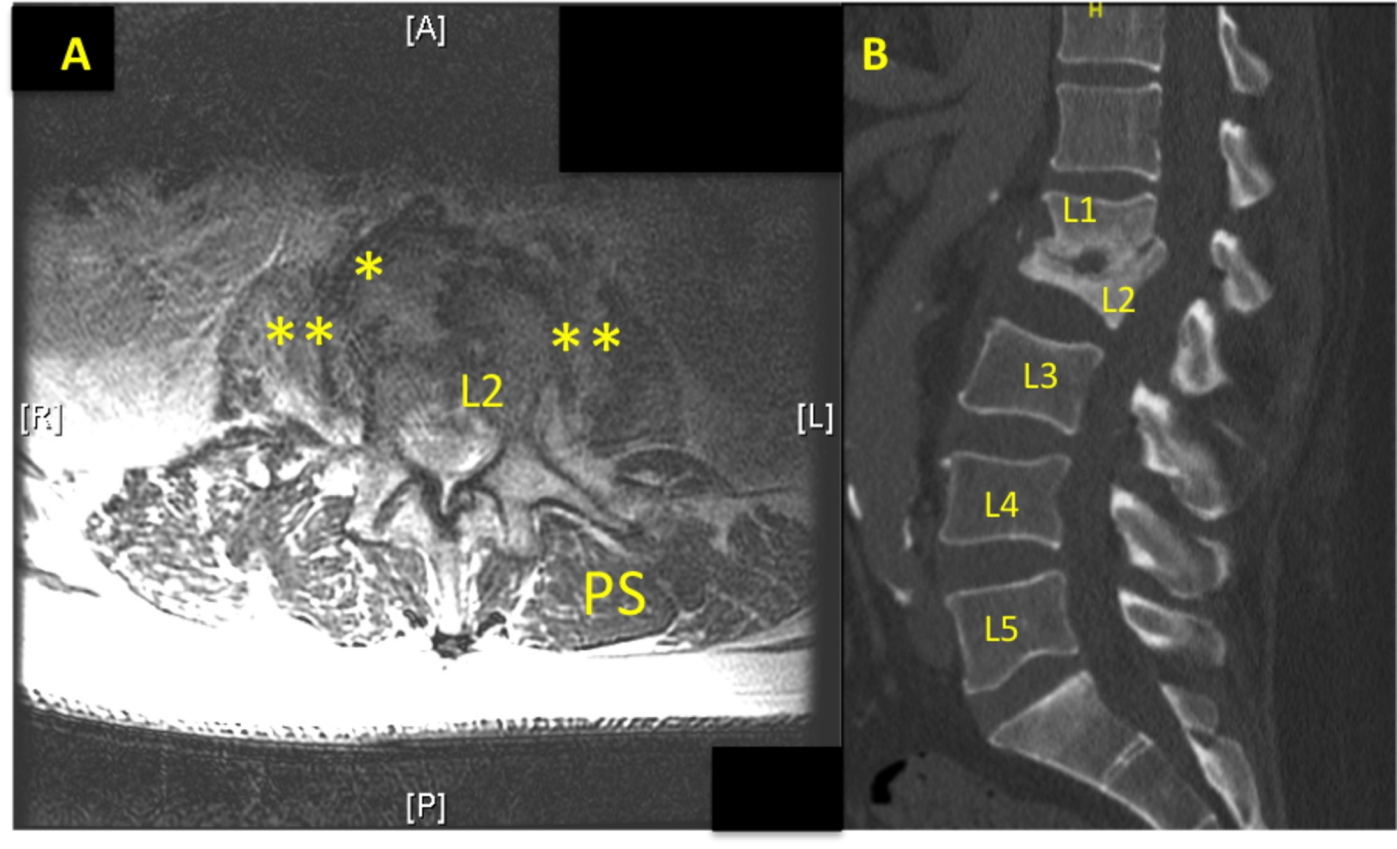
Re-admission MRI Lumbar and CT Lumbar Re-admission, Figure 2A: (MRI T2 wo contrast, axial): Non-diagnostic as the patient terminated the study after three sequences. Mild improvement in paravertebral abscess (yellow *). Psoas muscles (double yellow *). Paraspinal muscles (PS). Figure 2B (CT lumbar w contrast) Gibbus deformity at this level, discitis most likely reflecting progression of infection. Comparison to prior, progression of loss of vertebral body height of L2 and L1.

This case report involves a 62-year-old male with a prior history of epidural abscess and L1-L2 osteodiscitis, who was admitted to the ward because of progressively worsening low back pain. About four months prior (Figures 1A, 1B), the patient was treated via peripherally inserted central catheter (PICC) intravenous (IV) nafcillin (six week course) for methicillin-susceptible Staphylococcus aureus (MSSA) associated discitis in the L1/L2 vertebral region confirmed with CT guided aspiration. The patient reported doing well but two days prior to presentation, he started having severe low back pain again, along with subjective chills and fever. Magnetic resonance imaging (MRI) of the spine was ordered but the MRI study was limited due to the inability of the patient to tolerate being in supine position. The imaging that was obtained did show progression of loss of the L1-L2 vertebral bodies suspicious for osteomyelitis (Figures 2A, 2B). With a temperature of 100°F, elevated CRP > 9 mg/dL and lactic acidosis 2.8 mmol/L, vancomycin 1.25 gm IV and ceftriaxone 2 gm IV were given empirically for severe sepsis on admission (Day 0). The next day (Day 1), the Infectious Disease department was consulted and they recommended vancomycin 1.5 gm Q12H IV and cefepime 2 gm Q12H IV. However, the CBC labs showed pattern concerning for pancytopenia with WBC decreased to 2.5 thou/mm^3^, Hgb to 6.2 g/dL, Hct to 20.8%, and platelets to 82 thou/mm^3^ (Table 1). Although the patient was given IV NS fluids overnight, the degree and pattern of reduction was not consistent with hemodilution. Repeat CBC labs confirmed these values. Incidentally, on admission, his prothrombin time (PT) and international normalized ratio (INR) were elevated to 19.5 seconds and 1.72, respectively, but the patient stated that he consumes red yeast rice extract for cholesterol control, which also has anti-clotting properties. Lactate dehydrogenase (LDH) obtained on Day 1 was elevated to 929 unit/L and the total bilirubin on admission was 0.8 mg/dL.

**Table 1:** Complete Blood Count (CBC) Values Longitudinal

Table 1: CBC Values Longitudinal									
	Day 0 ED admit	Day 1	Day 2 (repeat labs)	Day 3	Day 4	Day 5	Day 6	Day 7	Day 8
Hemoglobin (g/dL)	8.0	8.3	6.2 (5.9)	6.9	7.6	7.2	7.3	7.5	7.6
Hematocrit (%)	27	28.3	20.8 (19.6)	22.4	24.2	22.6	23	23.6	24.7
White blood cells (thou/mm^3^)	4.6	3.9	2.5 (2.6)	5.7	8.0	7.3	6.7	6.9	7.0
Platelets (Thou/mm^3^)	86	126	82 (79)	90	98	94	86	147	194
Antibiotics	Vancomycin, Ceftriaxone	Vancomycin, Cefepime	Vancomycin, Cefepime	Nafcillin	Nafcillin	Nafcillin	Nafcillin	Nafcillin	Nafcillin
Events			Transfuse 2 units pRBC	Transfuse 1 unit pRBC					

Due to the patient's acute anemia on Day 2, a decision was made to transfuse two units of packed RBCs, which mildly improved the patient's Day 3 labs to WBC 5.7 thou/mm^3^, Hgb 6.9 g/dL, Hct 22%, and Plt 90 thou/mm^3^ (Table 1) with a D-Dimer of 741 ng/mL (Table 2). To evaluate for disseminated intravascular coagulation (DIC), fibrinogen and fibrin split products (FSP) were ordered on Day 2, but unfortunately, those labs were collected post transfusion. Nevertheless, fibrinogen was 544 mg/dL and FSP was 10-40 mcg/dL and the peripheral smear from Day 2 did not show schistocytes (Table 2). The Infectious Disease department recommended discontinuation of vancomycin and cefepime and resuming nafcillin 12 gm Q24H IV which the patient tolerated during prior admission. The patient received another transfusion on Day 3, which improved Day 4 labs of Hgb to 7.6 g/dL and Plt to 98 thou/mm^3^. Lactic acid decreased to 0.9 mmol/L; however, the patient continued to have fever spikes of temperature 101.6°F. On Day 3, his reticulocyte count was 0.4 (absolute 0.2), the reticulocyte index was 0.12 and haptoglobin was elevated to 315 mg/dL. The Hematology department was consulted and they ordered serum protein electrophoresis (SPEP), vitB12 and folate (both within normal limits). The SPEP result was obtained several days later, which yielded a kappa/lambda light chain ratio of 0.78 (WNL) and monoclonal immunoglobulin not detected by immune-fixation (Table 2). By Day 7 of monotherapy with nafcillin, CBC continued to improve with WBC of 6.9 thou/mm^3^, Hgb 7.5 g/dL, Hct 23.6%, and Plt 147 thou/mm^3 ^(Table 1). The blood cultures from admission were positive for MSSA staph and through interventional radiology guided spinal abscess aspiration, microbiology studies yielded a pan-sensitive staph organism. By Day 8, the patient had no fever spikes, but an esophagogastroduodenoscopy (EGD) was performed to rule out a gastrointestinal bleeding source since his fecal occult blood test was positive and due to the acute anemia soon after admission. EGD identified a chronic 2 cm duodenal ulcer, which was ablated, and colonoscopy was WNL. Due to the persistent bacteremia, transesophageal echocardiography (TEE) was also done, which ruled out valvular vegetation as a source for the MSSA bacteremia.

**Table 2:** Iron Profile/Hematology/SPEP Studies SPEP - serum protein electrophoresis, TIBC - total iron binding capacity, FeSat - iron saturation, retic - reticulocyte, LDH - lactate dehydrogenase, PEP - protein electrophoresis, IEP - immunoelectrophoresis.

Iron Profile/Hematology/SPEP Studies		
	Values (Day 4)	Reference range
Ferritin	97.1	24 – 336 ng/mL
Total iron	22	45 – 182 mcg/dL
TIBC	298	250 – 450 mcg/dL
FeSat	8	20 - 55 %
	Values (Day 3)	Reference range
Fibrinogen	544	150 – 400 mg/dL
Fibrin degradation products	10-40	<10 mcg/dL
D-dimer	741	<460 ng/mL
Retic count	0.4	0.6 - 2.2%
Haptoglobin	315	30-200 mg/dL
LDH	929	313 – 618 units/L
	Values (Day 4)	Reference range
Alpha-2-globulins	0.85	0.6 – 1.0 g/dL
Beta globulins	0.74	0.7 – 1.2 g/dL
Gamma globulins	0.63	0.7 – 1.6 g/dL
PEP interpretation	Hypogammaglobulinemia and hypoalbuminemia	
IFE kappa light chain	26.5	3.3 – 19.4 mg/L
IFE lambda light chain	33.9	5.7 – 26.3 mg/L
Kappa/lambda ratio	0.78	0.26 – 1.65
IEP interpretation	Monoclonal immunoglobulin is not detected by immunofixation	

## Discussion

This case presents a unique adverse reaction to vancomycin associated with both agranulocytosis and hemolytic anemia. Although the patient was exposed to vancomycin, ceftriaxone and cefepime, vancomycin was used the longest for three days. Vancomycin is known to have a 2% to 3% chance of being associated with neutropenia, and a less than 1% chance of causing thrombocytopenia [2]. Taking into consideration the Naranjo [3] adverse drug reaction (ADR) probability scale, there is a probable association between vancomycin and neutropenia [4,5] as well as between vancomycin and thrombocytopenia [2,6]. The Naranjo ADR probability scale ranges from doubtful to possible, probable and definite. Although the agranulocytosis and reticulocyte index suggest a bone marrow etiology, the elevated LDH and FSP also point to a hemolytic process such as DIC or immune thrombocytopenia (Table 2). Interestingly, DIC may have been present prior to admission given the sepsis and initial coagulopathy with prothrombin time (PT) of 19 seconds, partial thromboplastin time (PTT) of 30 seconds, and international normalized ratio (INR) of 1.72. The elevated fibrinogen level can be explained due to the post transfusion state or acute phase reactant. However, the low reticulocyte index and pancytopenia suggest a global bone marrow suppression. Although the elevated fibrinogen of 544 mg/dL, D-Dimer of 741 ng/mL and FSP of 10-40 mcg/dL may be confounded by the blood transfusion, DIC can not be ruled out. Similarly, although the peripheral smear was negative for schistocytes, this is not sensitive or specific for DIC [7]. Heparin-induced thrombocytopenia was ruled out since the patient was not on heparin. Multiple myeloma was also ruled out given the SPEP/IPEP data (Table 2). Although EGD identified a duodenal ulcer, this lesion was chronic and not actively bleeding. Furthermore, the patient denied a recent history of hematemesis, hematochezia or melena.

Hence, in this case, the pancytopenia may be due to DIC related to sepsis or a combination of initial DIC, exacerbated by drug toxicity to either vancomycin, ceftriaxone or cefepime. As stated earlier, initial lab values support the possibility of the patient having developed pancytopenia secondary to DIC from sepsis prior to admission. For instance, DIC can cause anemia through red blood cell fragmentation, or the anemia can also be associated with anemia of chronic disease in the setting of sepsis [8] as the iron profile showed low total iron, low FeSat, low TIBC, and relatively low ferritin (Table 2).

Furthermore, sepsis is a major risk factor for thrombocytopenia as it is a feature in up to 98% of DIC cases with the platelet count < 50 thou/mm^3^ in approximately 50% [9]. Leukopenia of 3.9 thou/mm^3^ also meets systemic inflammatory response syndrome (SIRS) criteria with WBC <4 thou/mm^3^ (Table 1). However, one argument against sepsis and DIC being the sole factors for the pancytopenia is that the reticulocyte count and retic index are consistent with a hypoproliferative marrow or bone marrow suppression. One would expect a consumptive process such as DIC induced thrombocytopenia to be associated with a hyper-proliferative bone marrow. The etiology of his pancytopenia was further complicated by the fact that his bone marrow began to recover when he was placed on nafcillin monotherapy as well as when the sepsis improved on Day 4 with a lactic acid of 0.9 mmol/L.

The notion of antibiotic induced aplastic anemia is also still applicable in this case. Given his lab values, this patient certainly fits the definition of acquired aplastic anemia as characterized by anemia, neutropenia, and thrombocytopenia, with a hypo-cellular bone marrow. Acquired, drug-induced aplastic anemia is an idiosyncratic reaction, with unpredictable severity and time to recovery. It has been estimated that 50% of aplastic anemia cases are acquired in nature, but a definitive causative agent cannot be identified in most cases [9]. The three major etiologies of acquired aplastic anemia include direct toxicity, metabolite-driven toxicity, and immune-mediated mechanisms [9]. For instance, a 2018 case report by Gniadek and a 2007 clinical report by Drygalski describe a mechanism of immune thrombocytopenia involving vancomycin dependent platelet reactive antibodies [6,10]. Pancytopenia has also been described to be more likely with both slow and fast rates of infusion as well as lengthy duration of vancomycin infusion [1,4]. Aplastic anemia has been identified in observational studies involving medications such as carbamazepine, methimazole, phenytoin and sulfonamides, whereas chloramphenicol, dapsone and lithium have been mentioned in case reports [9]. In this case, the patient was exposed to vancomycin, cefepime and ceftriaxone before completing his antibiotic course with nafcillin monotherapy.

## Conclusions

This case presents a unique incidence of pancytopenia in that the etiology appeared to involve elements of DIC related to sepsis as well as acquired aplastic anemia. Fortunately, the patient did not require high dose steroids or advanced therapeutics such as intravenous immunoglobulin infusion therapy (IVIG) or plasmapheresis as his hematologic function normalized after consolidating IV antibiotics to nafcillin. The objective of this case report is to emphasize early recognition of pancytopenia through relevant basic labs and hematologic workup. If the patient is undergoing IV antibiotics treatment, the antibiotics will have to be assessed carefully and his allergy list updated if an antibiotic is found to be associated with an adverse drug reaction.

## References

[1] Kaufman DW, Kelly JP, Issaragrisil S (2006). Relative incidence of agranulocytosis and aplastic anemia. Am J Hematol.

[2] Zenon GJ, Cadil RM, Hamill RJ (1991). Vancomycin-induced thrombocytopenia. Arch Intern Med.

[3] Naranjo CA, Busto U, Sellers EM (1981). A method for estimating the probability of adverse drug reactions. Clin Pharmacol Ther.

[4] Gupta S, Sharma S, Menon N, Ahuja S, Dahdouh M (2016). Case report of vancomycin induced pancytopenia. Rev Soc Bras Med Trop.

[5] Black E, Lau TTV, Ensom MHH (2011). Vancomycin-induced neutropenia: is it dose- or duration-related?. Ann Pharmacother.

[6] Drygalski AV, Curtis BR, Bougie DW (2007). Vancomycin-induced immune thrombocytopenia. N Engl J Med.

[7] Levi M, Toh CH, Thachil J, Watson HG (2009). Guidelines for the diagnosis and management of disseminated intravascular coagulation. Br J Haematol.

[8] Goyette RE, Key NS, Ely W (2004). Hematologic changes in sepsis and their therapeutic implications. Semin Respir Crit Care Med.

[9] Rao KV (2014). Chapter 24. Drug-induced hematologic disorders. Pharmacotherapy: A Pathophysiologic Approach 9e.

[10] Gniadek TJ, Arndt PA, Leger RM, Zydowicz D, Cheng EY, Zantek ND (2017). Drug induced immune hemolytic anemia associated with anti-vancomycin complicated by a paraben antibody. Transfusion.

